# Reverse-Polarization High-Performance Layer Electrochromatography—A New Approach to Anion Separation

**DOI:** 10.3390/ijms24119389

**Published:** 2023-05-28

**Authors:** Radosław Łukasz Gwarda, Tadeusz Henryk Dzido

**Affiliations:** Department of Physical Chemistry, Faculty of Pharmacy, Medical University of Lublin, 4a Chodźki Str., 20-093 Lublin, Poland; tadeusz.dzido@umlub.pl

**Keywords:** high-performance layer electrochromatography, overpressured-layer chromatography, anion separation, reverse-polarization electrochromatography, separation against electroosmotic effect

## Abstract

High-performance layer electrochromatography (HPLEC) combines the advantages of overpressured-layer chromatography (OPLC) and pressurized planar electrochromatography (PPEC) while overcoming some of their limitations. HPLEC equipment can work in various HPLEC, OPLC, and PPEC modes. The equipment enables HPLEC analysis also with an electroosmotic effect directed against the hydrodynamic flow of the mobile phase. The change in the electric field direction in the separation system does not result in a change in either the direction of the mobile phase flow or the direction of solute migration. The hydrodynamic flow generated by the pump dominates the electroosmotic effect and enables separation against the direction of the latter. Reversed-polarization HPLEC may be advantageous for the analysis of anionic compounds, as it facilitates faster and more selective separation than OPLC performed in similar conditions. This separation mode provides a new possibility to develop and optimize separation methods by performing separation against the electroosmotic effect and without need of any modification of the adsorbent surface. A drawback of this separation mode is the increase in the backpressure at the mobile phase inlet and the limitation of the mobile phase flow rate. Currently, contrary to the single-channel mode, multi-channel reverse-polarity HPLEC still requires some technical and methodological improvements.

## 1. Introduction

In our previous papers, we have presented new prototype equipment for high-performance (high-pressure) layer electrochromatography (HPLEC) [1] and some preliminary results of examples of separations in various working modes [2]. The new separation technique is a combination of overpressured-layer chromatography (OPLC) [3,4,5,6,7] and pressurized planar electrochromatography (PPEC) [8,9,10,11]. It overcomes the limitations of both these techniques. Contrary to PPEC, the flow of the mobile phase in HPLEC can be freely optimized using a high-pressure pump. The composition of the mobile phase can be optimized to obtain the required retention, without taking care of its influence on the electroosmotic effect. This is practically impossible in PPEC systems. On the other hand, voltage can be used to freely change the selectivity of separation of ionizable compounds, while its influence on electroosmotic flow does not have to be really taken under consideration. The use of voltage can, additionally, facilitate mobile phase flow through the adsorbent layer, thus decreasing the backpressure and allowing an increase in the limit of the overall flow velocity. These capabilities of HPLEC are clearly advantageous in comparison to OPLC. In addition to the fully controlled flow of the mobile phase, HPLEC also shows other advantages characteristic for column/capillary chromatography techniques, such as full automation, full equilibration of the separation system, and high performance (high flow of the mobile phase). Some other capabilities of our technique, such as simultaneous separation of multiple samples as well as the use of various sample application and detection methods, are characteristic advantages. All this makes our prototype equipment able to work in many combinations of sample application and detection modes (fully online, fully offline, online + offline, or offline + online) and with various separation techniques (OPLC, PPEC, HPLEC, single- or multichannel separation) [1,2].

In our previous paper [2], we have shown some examples of comparisons of OPLC and HPLEC separations. The experiments were carried out in various working modes and in various separation conditions. In turn, all separations with the use of voltage were carried out with a standard setup of system polarization, i.e., an anode was placed on the inlet side of the separation system, while the cathode was located on the outlet side. A standard set of solutes of various properties (polarity, pKa) was used (mainly food dyes; azo dyes). The electrophoretic movement of cations was directed according to the hydrodynamic mobile phase flow, the same as the generated electroosmotic flow. At a relatively low pH of the mobile phase (3.0), the solutes being separated were mainly in the cationic form. This resulted in appropriate acceleration of their movement and separation process. In addition, the advantage was the facilitation of the mobile phase flow by the electroosmotic effect, resulting in a reduction in backpressure on the inlet side of the separation system.

**Figure 1:**
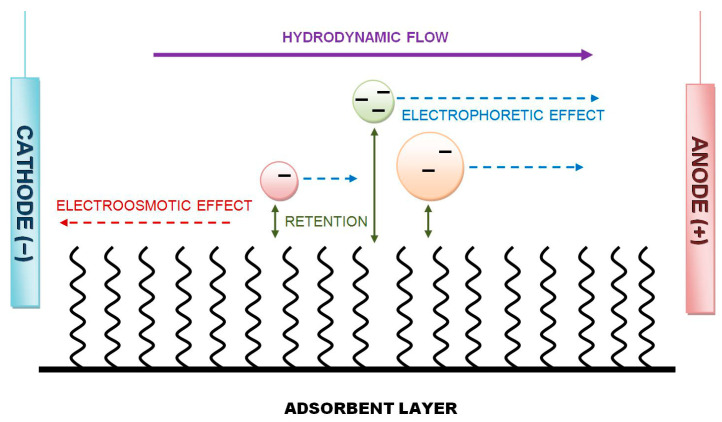
General scheme and mechanism of separation in reverse-polarization HPLEC.

Our HPLEC equipment has been designed to also work in a reverse-polarization mode (with a cathode placed near the inlet side of the separation system and an anode located near the outlet side), where only a simple change in the electrode connection is needed [1]. This means that the direction of the total flow of the mobile phase is not changed (contrary to the capillary electrophoresis/electrochromatography techniques CE/CEC), and the direction of solute movement is the same as well. The general scheme and principle of separation in reverse-polarization HPLEC is shown in Figure 1.

The only changes expected were the electroosmotic effect directed against the hydrodynamic flow of the mobile phase and the accelerated movement of anions (instead of cations). A similar approach has not been described so far. Inversion of the polarization in CE/CEC systems requires modification of the capillary/adsorbent surface to maintain the direction of electroosmotic flow [12]. Reverse-polarization HPLEC is expected to overcome this limitation, as the mobile phase flow depends on a high-pressure pump instead of an electroosmotic effect.

The aims of our current work are as follows:

- investigation of reverse-polarization HPLEC separation mode with our prototype equipment, using a set of solutes similar to those used before in normal-polarization HPLEC [2];
- comparison of the results with OPLC in similar separation conditions;
- investigation of the influence of the change in the electric field direction, especially the influence of the redirection of the electroosmotic effect against the hydrodynamic flow of the mobile phase on the total flow profile and direction, backpressure and velocity limit of the mobile phase flow, velocity and direction of solute migration, and overall performance of separation;
- evaluation of any possible side effects of the approach;
- discussion of the advantages and disadvantages of the reversed-polarization HPLEC mode and its practical potential.

## 2. Results and Discussion

In our previous experiments, acidic mobile phases were used for the separation of a standard set of solutes (mainly azo dyes) in various modes of normal-polarization HPLEC [2]. The solutes being separated were mainly in the cationic form. This resulted in an acceleration of their movement in the electric field and, thus, an acceleration of the whole separation process. Here, we chose ammonium acetate as the mobile phase additive to maintain pH near the neutral value. This allowed a similar set of solutes to form negatively charged ions with high electrophoretic mobility toward the anode. The use of ammonium acetate resulted from a compromise between efficiency of separation (peak shape) and electric current (separation system heating, gas baubles formation). The pH of the mobile phase was not increased to higher values to avoid issues with adsorbent stability.

### 2.1. Advantages of Our Equipment and Reverse-Polarity HPLEC

**Figure 2:**
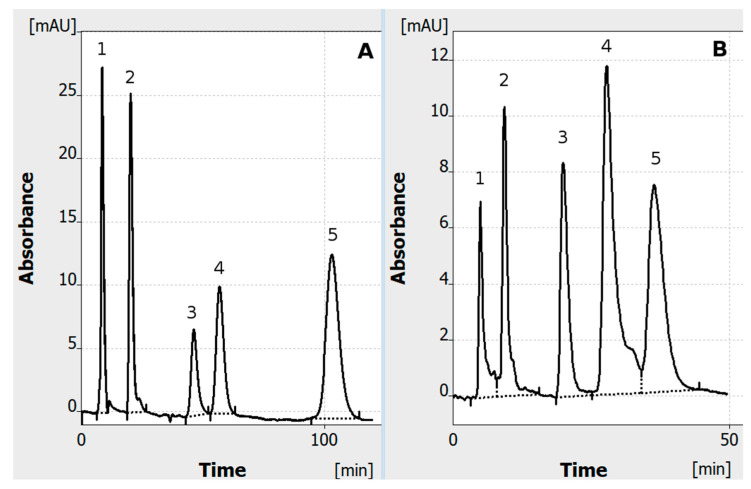
Comparison of single-channel OPLC (**A**) and reverse-polarization HPLEC (**B**) of the dye mixture with the electroosmotic effect directed against the total flow of the mobile phase and migration of solutes. In total, 1 µL of the mixture was applied to the chromatographic plate in the on-line sample injection mode. Separation system used: HPTLC RP-18 W chromatographic plates; water/methanol (7/3 *v*/*v*) with the addition of 40 mM ammonium acetate (final concentration) as the mobile phase; total mobile phase flow 0.2 mL/min; voltage 0 kV (**A**) and −4 kV (**B**); temperature 25° C, cushion pressure 100 bar; mobile phase backpressure 83 bar (**A**), 90 bar (**B**). Dye mixture: 1—tartrazine 0.0125% (*w*/*v*); 2—sunset yellow 0.025% (*w*/*v*); 3—allura red 0.025% (*w*/*v*); 4—% o-nitroaniline—0.0125% (*w*/*v*); 5—azorubine; 0.1 (*w*/*v*).

The results show (Figure 2) that the test dye mixture can be successfully separated by both OPLC and reverse-polarization HPLEC. The comparison of separation carried out with these two techniques shows that the mixture can be separated over two times faster with HPLEC than with OPLC. This is due to the share of the electrophoretic effect in the separation process, as well as due to the change in the adsorbent properties during its conditioning with high voltage.

**Figure 3:**
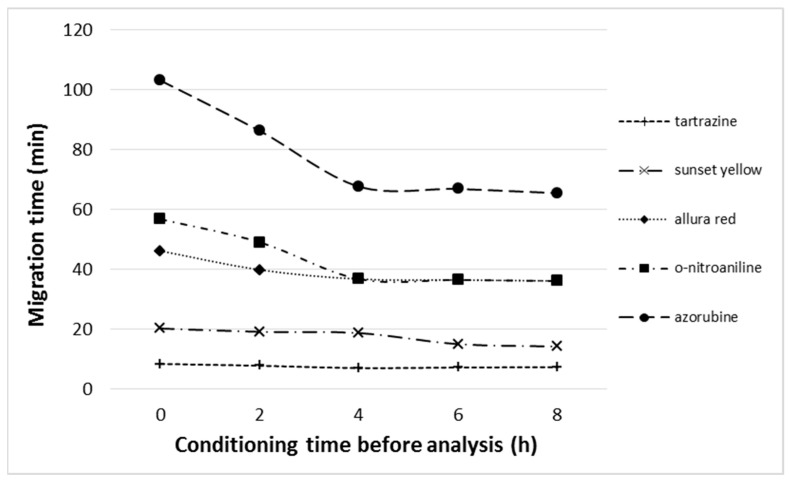
Relationship: solute migration time vs. adsorbent conditioning time before the analysis. Separation system used: HPTLC RP-18 W chromatographic plates; water/methanol (7/3 *v*/*v*) with the addition of 40 mM ammonium acetate (final concentration) as the mobile phase; total mobile phase flow 0.2 mL/min; temperature 25 °C; cushion pressure 100 bar.

Our results show that separation with OPLC is highly unrepeatable. Figure 3 proves that properties of the adsorbent changes and retention considerably decreases as a result of long flow of the mobile phase through the adsorbent layer—either during the following OPLC analyses or long conditioning. This occurs despite the baselines being smooth and stable, and the separation system seems to be equilibrated. This problem does not occur in HPLEC, where conditioning with high voltage is used. HPLEC analyses are quite repeatable (see Table 1).

This suggests that the decrease in retention is related to the removal of some ionic residues from the adsorbent layer. This purification process is faster with the use of high voltages because of the electrophoretic effect. In our previous paper, we suggested that the use of high voltages may accelerate/enhance conditioning of the adsorbent layer and equilibration of the separation system [2]. In another paper, we showed that chromatographic plates may contain a significant amount of ionic residues [13]. These observations suggest that standard, commercially available chromatographic plates may not be suitable for multiple analyses in fully online OPLC systems. On the other hand, after the conditioning with high voltages, the same plates can also be used to obtain repeatable results of multiple analyses in an OPLC system (Table 1). This shows yet another additional advantage of our equipment—it can be used for fast conditioning of chromatographic plates for OPLC analyses.

**Figure 4:**
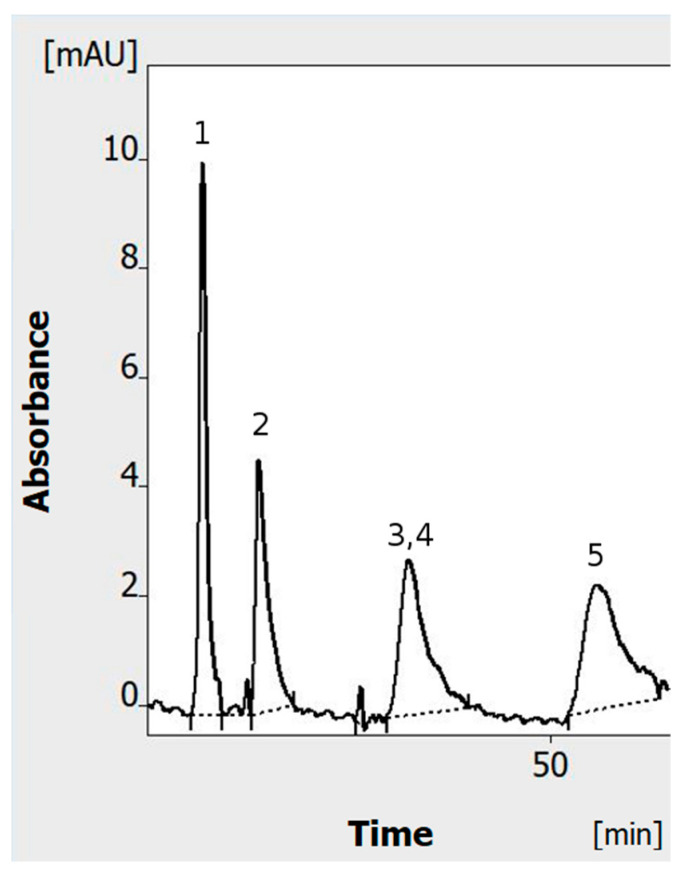
OPLC of the dye mixture after adsorbent conditioning with high voltage for 1 h (as described in Materials and Methods for HPLEC experiments). In total, 1 µL of the mixture was applied to the chromatographic plate in on-line sample injection mode. Separation system used: HPTLC RP-18 W chromatographic plates; water/methanol (7/3 *v*/*v*) with addition of 40 mM ammonium acetate (final concentration) as the mobile phase; total mobile phase flow 0.2 mL/min; temperature 25° C, cushion pressure 100 bar. Dye mixture: 1—tartrazine 0.0125% *w*/*v*; 2—sunset yellow 0.025% *w*/*v*; 3—allura red 0.025% *w*/*v*; 4—% o-nitroaniline—0.0125%*w*/*v*; 5—azorubine; 0.1 *w*/*v*.

After the adsorbent layer is fully conditioned, its properties are clearly different in comparison with those of its factory state. This considers not only the overall decrease in retention, but also changes in the retention mechanism, and, in effect, the selectivity of separation. Figure 3 shows that allura red and o-nitroaniline, which could be previously separated in an OPLC system (Figure 2A), have the same retention after full conditioning of the adsorbent. They cannot be separated and coelute as a single peak (Figure 4).

The chromatogram shows that the overall retention decreases (in comparison with Figure 1A) as well as the time of analysis. Still, the migration time of the solutes and overall time of analysis (65 min) is longer than in those in the HPLEC (45 min, Figure 1B). In the latter, the hydrodynamic flow of the mobile phase has the same direction as the electrophoresis of anions. Their migration velocity clearly increases in the electric field. The electrophoretic effect also influences the selectivity of separation, allowing us to separate the compounds that cannot be separated in a similar OPLC system. Table 1 shows that both reverse-polarization HPLEC and OPLC (with properly conditioned adsorbent) allowed us to obtain relatively repeatable results. RSD of the migration time for most of the separated compounds was between about 1% and 3%. It was slightly higher (3.45%) for azorubine in the OPLC system. This still could possibly result from some system equilibration issue. Anyway, all the repeatability results are comparable (or even slightly better) to those reported before for thin-layer chromatography and pressurized planar electrochromatography systems [8,9].

Our results suggest that the change in the direction of the electric field in the HPLEC system can be useful and advantageous in anion analysis. Anions can be successfully separated against the direction of the electroosmotic effect. In contrast to the CE/CEC techniques, there is no need here to modify the silica surface to obtain the change in the direction of the electroosmotic flow. Our results show that the hydrodynamic pressure dominates the electroosmotic effect. Importantly, the opposition of the hydrodynamic flow and the electroosmotic effect seems not to result in any disorder of the mobile phase flow, which could be theoretically suspected. We did not observe any chromatogram distortions or any other unpredictable effects related to the reverse-polarization of the separation system. Naturally, some fluctuations of the baseline (related to use of the high voltage) could be observed, but those were comparable to the fluctuations observed with use of the normal-polarization setup [1,2]. This shows that our equipment provides a new possibility to develop and optimize separation methods by performing separation against the electroosmotic effect. Such possibility was not reported before for any other equipment or separation technique.

A somewhat similar case was described before for pressurized CEC. In that case, however, a relatively low pressure (up to 50 psi) was opposite to the electroosmotic flow, which remained the main driving factor of the separation. Thus, the direction of the separation was the same as the direction of the electroosmotic effect, but opposite to the applied pressure [14].

We emphasize that the aim of our work is not to develop any specific separation methods, nor their comparison. All the analyses presented here can be further optimized—this should probably be conducted for HPLEC and OPLC systems separately. The optimization should concern not only the separation system itself, but also some working parameters, such as the flow ratio of sample injection and/or detection vs. total mobile phase flow, flow of electrode compartment rinsing, etc. Moreover, the results can surely be improved with further development of the equipment and with its precise and professional manufacturing. It should be considered that they were obtained with the first prototype HPLEC equipment elaborated in our department (not with a ready-to-sell commercial product). There is no sense, nor reason, to compare them with any results obtained with any commercial analytical equipment. The only purpose of the results we present is to show the unique potential of the HPLEC technique and the new analytical approach—electrochromatographic separation against the electroosmotic effect. Naturally, anions can be successfully separated/analyzed using CE, CEC, or HPLC [12,15,16,17,18,19], probably easier/faster/more repeatable, etc. (at this point). Still, we believe that the unique potential, versatility, ease of changing operational modes, and high throughput may result in high competitiveness of HPLEC if it can reach its final, professional, and commercial shape.

### 2.2. Drawbacks and Limitations of Reverse-Polarization HPLEC

There are some drawbacks and limitations related to the reverse-polarization operational mode of our HPLEC equipment. First, the direction of the electroosmotic effect against the hydrodynamic pressure of the mobile phase resulted in an increase in the backpressure at the mobile phase entry site. Here, the backpressure increased from 83 bar in the OPLC mode to 90 bar with –4 kV applied in the HPLEC mode.

**Figure 5:**
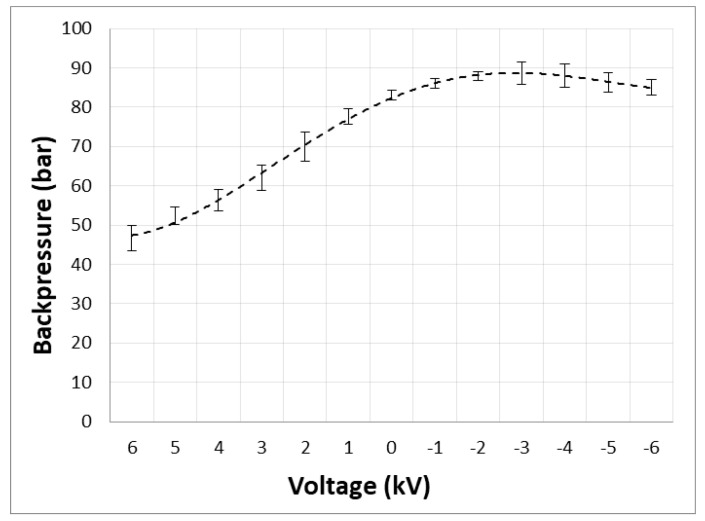
Relationship: backpressure vs. separation system polarization. Reverse-polarization (electroosmotic effect turned against the hydrodynamic flow of the mobile phase) is indicated as negative voltage values. Separation system used: HPTLC RP-18 W chromatographic plates; water/methanol (7/3 *v*/*v*) with the addition of 40 mM ammonium acetate (final concentration) as the mobile phase; total mobile phase flow 0.2 mL/min; temperature 25 °C; cushion pressure 100 bar.

Figure 5 presents the relationship of backpressure vs. voltage applied to the separation system. It proves that the backpressure changes to a greater extent in the normal-polarization mode (electroosmotic effect supports mobile phase flow) than in the reverse-polarization mode. In the range from 0 kV to 6 kV, the backpressure decreases almost two times. In the range from 0 kV to −4 kV, it slightly increases (by about 8%); then, it decreases very slightly in the range from −4 kV to −6 kV, close to the base value (at 0 kV). In theory, a similar decrease and increase in the backpressure, depending on the polarization mode, could be expected. The discrepancies observed may result from two sources. First, the cooling system may be not efficient enough to counterbalance the Joule’s heat formation in the adsorbent layer. The increase in the temperature may lead to a decrease in the mobile phase viscosity and, hence, to a decrease in the backpressure for both the normal- and reverse-polarization modes. This effect may be more pronounced at the higher voltages used. This may be supported by the results presented in Figure 5. At high voltages, the decrease in the backpressure related to the decrease in the mobile phase viscosity may even dominate the increase in the backpressure related to the counteraction of the electroosmotic effect. On the other hand, the electroosmotic effect can change the mobile phase flow ratio between the adsorbent layer and the venting valve of the inlet side electrode compartment. This effect was also confirmed by our experiments, yet no specific flow ratio was measured/recorded at the specific voltage value due to technical difficulties (need of use of the safety box during the experiments with high voltage—no access to outlet/venting tubing). Certainly, the flow ratio should be set for specific experimental conditions, including the polarization mode and voltage used. Notably, the use of a lower buffer concentration than that presented above may lead to a further increase in the electroosmotic effect with a decrease in the Joule’s heat formation. This may change the relationship presented in Figure 5.

The results show that the opposition of the electroosmotic effect and the hydrodynamic flow of the mobile phase may result in some limitation of the maximum flow in this separation mode. This is related to the fact that the pressure of the mobile phase in the separation system cannot exceed the pressure in the cushion pressurizing the adsorbent layer. Another difficulty noticed is related to the stability of the electrochromatogram baseline. As we mentioned in our previous paper [2], the use of voltage results in the deterioration of the baseline stability, especially in the multichannel separation mode. Here, this effect seems to be even more pronounced. In the single-channel reverse-polarization HPLEC (with a constant flow of the mobile phase from the sample pump during the whole separation process), only a slight deterioration of the electrochromatogram baseline can be observed. However, our attempts at multichannel separation in reverse-polarity HPLEC yielded a more unstable (than in normal-polarity mode) chromatogram baseline. This makes it difficult to obtain reliable and repeatable analyses in the multichannel working mode, at least at such a low concentration of solutes. Naturally, an increase in the solute concentration may be supposed to improve the overall electrochromatogram shape (lower influence of the baseline fluctuation); however, this would not really solve the problem.

It must be mentioned that, in the normal-polarization mode, our HPLEC equipment was able to work constantly over 24 h. In the reverse-polarity mode, the baseline distortions seemed to be even more pronounced with the length of the equipment work with the voltage applied. In an extreme case, fluctuations in the electric current may also be observed. This clearly suggests that the problem may result from the formation of gas bubbles, probably mainly in the electrode compartments. In our HPLEC equipment, the flow of the mobile phase and gas diffusion from the electrode compartment to the mobile phase inlet trough is restricted. However, this restriction seems not to be efficient enough to fully prevent the development of gas bubbles in the part of the adsorbent layer in which the separation occurs. Possible local changes in temperature may facilitate the formation of bubbles, as the chromatographic plate is cooled only from the bottom side. It should be emphasized that, here, we present only preliminary results of the reverse-polarization HPLEC mode. The electrode compartments in our equipment are constantly vented and rinsed by a fresh mobile phase. It is possible that optimization of the electrode compartment rinse (flow velocity/pressure restriction) along with optimization of other working parameters, e.g., temperature, voltage, total flow, and pressure of the mobile phase, pressure in the pressurizing cushion, mobile phase composition, etc., may result in a reduction in the undesirable effects described. Such an approach, however, would be a limitation of the working parameters mentioned above; thus, it seems not to be the best solution to the problem. In our opinion, further and more effective restriction of flow/diffusion between the electrode compartment and the mobile phase inlet trough should give better results. We believe that modification of our HPLEC chamber in this respect should be one of our priorities. Naturally, the issue of reverse-polarization HPLEC requires further detailed investigation. The applicability of this working mode in practice needs to be further investigated as well. Nonetheless, the results described here suggest that reverse-polarization HPLEC may be advantageous in the analysis of anionic compounds, especially if the reliability and repeatability of multichannel separations could be improved and the analysis throughput could be increased.

## 3. Materials and Methods

### 3.1. Equipment and Chemicals

- The prototype HPLEC equipment was designed and constructed in the Department of Physical Chemistry, Medical University of Lublin, and was presented and described in our previous paper [1]. For our current experiments, reverse-polarization of the separation system was set so that the cathode was placed near the inlet side of the separation chamber and the anode was placed in the outlet electrode compartment near the sample outlet tubing. All other elements of the equipment were set in a standard way, as described before [1].
- The external pressure supply unit was purchased from P.W. Rafkop (Lubartów, Poland). A high-voltage power supply EV262 was bought from Consort (Turnhout, Belgium). Two quaternary HPLC pumps Azura P6.1L, an automatic six-channel selection valve Azura V2.1S, an autosampler Azura AS6.1L, and six UV detectors Azura UVD 2.1S (with analytical flow cells—path length 3 mm, capacity 2 µL) were bought from Knauer (Berlin, Germany). A circular thermostat AD07R-20 was bought from PolyScience (Niles, MI, USA).
- Certified analytical standard dyes were purchased from the Institute of Dyes and Organic Products (Zgierz, Poland). Ammonium acetate (analytical grade) and methanol (for HPLC—super gradient) were purchased from POCH (Gliwice, Poland). Water used in all experiments was purified using an HLP demineralizer from Hydrolab (Gdańsk, Poland). Glass-backed HPTLC RP-18 W plates were purchased from Merck (Darmstadt, Germany).
- A standard set of solutes was used (mainly food dyes; azo dyes), similar to the one used before in normal-polarization HPLEC [2] (indigotine was replaced by tartrazine, as properties/migration time of both are similar). The dye mixture was prepared by mixing and diluting of 0.5% dye stocks solutions (*w*/*v*). All stocks and the final mixture were prepared in a water/methanol solution (1/1 *v*/*v*). Finally, a dye mixture with the following composition was used: 1—tartrazine 0.0125% (*w*/*v*); 2—sunset yellow 0.025% (*w*/*v*); 3—allura red 0.025% (*w*/*v*); 4—azorubine; 0.1% (*w*/*v*); 5—o-nitroaniline—0.0125% (*w*/*v*). Except o-nitroaniline, all the solutes were azo dyes with strong acidic sulfo groups.

### 3.2. Experimental Conditions

- The experiments were performed in the single-channel mode at the temperature of 25 °C, applying 100 bar pressure to the cushion pressurizing the adsorbent layer. The mobile phase was a mixture of water/methanol (7/3 *v*/*v*) with the addition of 40 mM ammonium acetate (final concentration). The HPLEC experiments were performed with 4 kV polarization voltage with the cathode on the inlet side of the separation system.
- Before separation, the adsorbent layer was washed (conditioned) with the mobile phase until a completely flat baseline was obtained (for at least 1 h). The composition and flow of the mobile phase were the same as those used in the experiments. Before HPLEC separation, the voltage gradient was also applied from 0 V to the final value used for separation for 1 h. After the conditioning, chromatographic plates were used for multiple analyses. All statistics were made on the basis of five independent analyses (using the single or multiple chromatographic plates, respectively).
- In the experiments, a 1 µL sample was applied using the autosampler. The sample was injected into the stream of the mobile phase and pumped by the sample pump. The sample in the stream of the mobile phase was applied to the chromatographic plate. The mobile phase flow provided by the sample pump (after sample application) was sustained during the whole experiment.
- For both the OPLC and HPLEC experiments, the venting valve of the inlet side electrode compartment was opened, facilitating a constant wash of the electrode with the mobile phase. The flow through the venting valve was restricted to about 2% of the total mobile phase flow. Backpressure measured at the mobile phase inlet was 83 bar for OPLC and 90 bar for HPLEC separations.
- Separated solutes were detected at the 256 nm wavelength with a sampling rate of 2 Hz and time constant of 1 s. The migration time (t_M_) was established automatically by the software. The identification of the solutes was based on the comparison between t_M_ of mixture components and single standards separated in the same conditions.
- Most of the HPLEC equipment modules were controlled by a computer with ClarityChrom software 7.4.1.88. Only the high-voltage power supply, the pressure supply unit, and the circular thermostat were programed independently.

## 4. Conclusions

The test mixture of anionic dyes was successfully separated using our prototype equipment in reversed-polarization HPLEC working mode. Reliable, repeatable separation was not possible with OPLC due to the problems related to properties of the adsorbent—HPTLC RP-18 W plates from Merck are not suitable for fully online OPLC. HPLCE equipment used with high voltages can be used to considerably accelerate the conditioning of chromatographic plates for OPLC analyses. The comparison of both separation techniques showed that reverse-polarization HPLEC may be advantageous for analysis of anionic compounds. This was confirmed by the shorter time of analysis and the better selectivity of separation in comparison to OPLC. The change in the separation system polarization did not result in a change in the direction of the mobile phase flow or the direction of separation. The hydrodynamic pressure generated by the pump dominated the electroosmotic effect. In effect, HPLEC provides a new possibility to develop and optimize separation methods by use of the reverse-polarization working mode. This mode allowed us to perform separation against the electroosmotic effect and does not require any modification of the capillary/adsorbent surface (in contrast to CE/CEC). Similar possibilities were not reported before for any other equipment or separation technique. A drawback of the reversed-phase HPLEC mode is the increase in the backpressure at the mobile phase inlet, which results in the limitation of the maximum mobile phase flow that can be used. In multichannel reverse-polarity HPLEC, the baselines of simultaneously developed electrochromatograms are more unstable and fluctuating in comparison to the normal-polarization multichannel HPLEC mode. This probably results from the formation of gas bubbles in the separation system and affects the repeatability of multichannel analysis. To prevent this, the best solution seems to be to modify the HPLEC equipment to efficiently prevent the mobile phase flow and gas diffusion from the electrode compartment to the mobile phase inlet trough.

## Figures and Tables

**Table 1:** Repeatability of the migration time (RSD).

	OPLC after Conditioning with Voltage, Single Plate	HPLEC Single Plate	HPLEC between Plates/Days
**tartrazine**	1.37%	1.00%	1.35%
**sunset yellow**	2.02%	2.46%	3.08%
**allura red**	1.01%	1.20%	3.03%
**o-nitroaniline**	1.01%	1.07%	2.15%
**azorubine**	3.45%	2.08%	2.46%

## Data Availability

Not applicable.

## Sample Availability

All samples and other chemicals are commercially available.
